# The oral fungal mycobiome: characteristics and relation to periodontitis in a pilot study

**DOI:** 10.1186/s12866-017-1064-9

**Published:** 2017-07-12

**Authors:** Brandilyn A. Peters, Jing Wu, Richard B. Hayes, Jiyoung Ahn

**Affiliations:** 1 Division of Epidemiology, Department of Population Health, New York University School of Medicine, 650 First Ave, New York, NY 10016 USA; 2 NYU Perlmutter Cancer Center, New York University School of Medicine, New York, NY USA; 3 Present address: Microbiology section, New York City Public Health Laboratory, New York, NY USA

**Keywords:** Mycobiome, Oral, Periodontal disease, Periodontitis, Fungus, ITS

## Abstract

**Background:**

The oral fungal microbiome (mycobiome) is not well characterized, particularly in relation to oral diseases such as periodontal disease. We aimed to describe and compare the oral mycobiome of subjects with and without periodontal disease.

**Results:**

We characterized the oral mycobiome in 30 adult subjects (15 with periodontal disease, 15 with good oral health) by sequencing the taxonomically informative pan-fungal internal transcribed spacer (ITS) gene in DNA extracted from oral wash samples. We observed at least 81 genera and 154 fungal species across all samples. *Candida* and *Aspergillus* were the most frequently observed genera (isolated from 100% of participants), followed by *Penicillium* (97%), *Schizophyllum* (93%), *Rhodotorula* (90%), and *Gibberella* (83%). *Candida* and *Aspergillus* were also the most highly abundant genera in the samples (median relative abundance = 21% and 44%, respectively). *Aspergillus niger* was the most highly abundant species in the samples (median relative abundance = 44%). We did not observe significant differences in overall oral mycobiome diversity or composition between participants with periodontal disease and participants with good oral health, nor did we observe significant differences in phylum through species level taxon relative abundance or carriage between the two groups. Genus *Candida*, previously associated with periodontal disease in culture-based studies, had higher median relative abundance in participants with periodontal disease (33.2%) compared to participants with oral health (2.2%), though the difference was not significant (*p* = 0.52). Additionally, within the periodontal disease group, median relative abundance of *Candida* increased with increasing number of permanent teeth lost (1–2 teeth lost: 3.2%; 3–4 teeth lost: 16.6%; ≥5 teeth lost: 73.9%; *p* = 0.11), though sample size was small for this analysis.

**Conclusions:**

In this first study comprehensively characterizing the oral mycobiome of adults with periodontal disease or good oral health, we observed trends of higher *Candida* abundance in participants with periodontal disease, and participants with greater tooth loss. Small sample size may have limited the power to detect significant associations. Larger studies including subgingival samples may further establish the core oral mycobiome in health, and relate it to periodontal disease.

## Background

The human oral cavity hosts a complex microbiome comprised of an estimated 600 bacterial species [1] and 100 fungal species [2]. While the role of oral bacteria in human health and disease is increasingly well characterized [3–5], the role of oral fungi remains largely uncharacterized [6], with the exception of opportunistic *Candida* infection (oral candidiasis) [7]. In 2010, Ghannoum et al. published a landmark study characterizing the “basal” oral fungal microbiome (mycobiome) in 20 healthy individuals, by sequencing the pan-fungal internal transcribed spacer (ITS) gene in DNA extracted from oral rinse samples [2]. They identified 74 culturable and 11 non-culturable genera, and 101 species [2]. This study was an important first step to describe the healthy oral mycobiome, paving the way for studies of the oral mycobiome in disease.

Periodontal disease, or periodontitis, is a chronic inflammatory disease of the periodontium, the tissues that surround and support the teeth [8]. It effects 10–15% of adults [9] and is the most common cause of tooth loss worldwide [8]. Periodontal disease is caused by a synergistic and dysbiotic dental plaque microbial community, with keystone pathogens such as *Porphyromonas gingivalis* initiating the disruption of tissue homeostasis [10]. However, little is known regarding the involvement of oral fungi in this disease. Several studies have reported increased subgingival colonization by yeasts, particularly *Candida albicans*, in chronic periodontitis patients compared to periodontally healthy subjects [11, 12]. However, these studies were limited by culture-based methods, which cannot fully characterize the diversity of the mycobiome due to non-culturables.

We characterized the oral mycobiome in 15 adults with periodontal disease, and 15 periodontally healthy adults, using ITS gene sequencing of oral wash samples. Here, we describe the characteristics of the oral mycobiome, and compare overall diversity and relative abundance of fungal taxa between oral health and periodontal disease groups.

## Methods

### Study population

The 30 participants in this analysis were selected as a pilot project from a larger study which enrolled 239 participants from Kips Bay Endoscopy Center in New York City (June 2012–August 2014). Eligible participants for the parent study were individuals 18 years or older (range: 29–86) who recently underwent colonoscopy, were able to read English, and were not on long-term antibiotics. The study was approved by the institutional review board of NYU School of Medicine, and all participants provided written informed consent for all study procedures, including questionnaires and oral wash sample collection.

For the current analysis, we selected 15 subjects with periodontal disease, and 15 periodontally healthy subjects. Subjects with periodontal disease were those who answered “yes” to the question: “Has your dentist told you that you have periodontal disease with bone loss?” on a paper questionnaire. Periodontally healthy subjects were those who answered “no” to this question, and also reported no tooth loss, no swollen, inflamed, or bleeding gums, and no recommendations from their dentist to see a periodontist for bleeding gums or loose teeth. All 30 subjects were white and former smokers.

### Oral wash samples

Participants were asked to swish vigorously with 10 ml Scope mouthwash (P&G) and expectorate into a tube, as has been done in previous large cohort studies [13, 14]. Participants collected their samples at home (not at a specific time during the day) and mailed them to NYU, where upon receipt they were immediately stored at −80 °C.

### Mycobiome assay

We extracted DNA from oral wash samples using the PowerSoil DNA Isolation Kit (Mo Bio, Carlsbad, CA) following manufacturer’s instructions. Barcoded amplicons were generated covering the ITS gene region using the ITS1-F/ITS2 primer pair, as previously described by Luan et al. [15] (ITS1-F: 5′ CTTGGTCATTTAGAGGAAGTAA 3′ and ITS2: 5′ GCTGCGTTCTTCATCGATGC 3′). We employed FastStart High Fidelity PCR system (Roche, IN) to prepare 50 μl PCR reaction volumes for each sample, containing 0.2 μM of forward and reverse primers, 200 μM of deoxynucleoside triphosphate (dNTP) mix, 5 μl of 10× FastStart High Fidelity reaction buffer, 2.5 U (0.5ul) FastStart High Fidelity enzyme, and sample genomic DNA. PCR reactions were run at 95 °C for 5 min followed by 35 cycles of 95 °C for 1 min, 53 °C for 45 sec, and 72 °C for 1 min, and a final extension at 72 °C for 7 min. PCR products were purified using Agencourt AMPure XP (Beckman Coulter Life Sciences, IN) and quantified using the Agilent 4200 TapeStation (Agilent Technologies, CA). Amplicon libraries were pooled at equimolar concentrations and sequenced on Illumina MiSeq with a 300-cycle (2 × 151 bp) reagent kit.

### Sequence read processing

Multiplexed paired-end sequence reads were joined at overlapping regions using fastq-join [16], as implemented in QIIME script *join_paired_ends.py* [17]. Joined sequence reads were then demultiplexed (i.e. separated) into individual samples based on sample-specific sequence barcodes, and poor-quality reads were excluded, using default parameters in QIIME script *split_libraries_fastq.py* [17]. The 8,607,862 quality-filtered reads (from *n* = 30 samples) were clustered into operational taxonomic units (OTUs) against the QIIME/UNITE reference sequence collection (alpha version 12_11), and assigned taxonomy, using QIIME script *pick_open_reference_otus.py* [17]. In this method, reads not matching the reference sequence collection are clustered into OTUs de novo (i.e. based on sequence similarity), and OTUs with <2 reads are discarded, leaving 8,568,701 reads (reads/sample mean ± SD = 285,623 ± 182,857; range = [13,206–762,616]) and 8493 OTUs.

### α-diversity

α-diversity (within-subject diversity) was assessed using richness, which is a count of the number of OTUs in each oral mycobiome, and evenness, which is a measure of how equal the abundances of the OTUs are in each oral mycobiome. These indices were calculated in 100 iterations for rarefied OTU tables (minimum: 1000 reads/sample, maximum: 13,000 reads/sample) using QIIME script *alpha_rarefaction.py*. We examined whether α-diversity (at 13,000 sequence reads/sample) differed between the two periodontal groups using the Wilcoxon rank-sum test.

### β-diversity

β-diversity (between-subject diversity) was assessed using the Jaccard index, which considers similarities in OTU presence/absence between samples, and the Jensen-Shannon divergence (JSD), which considers similarities in OTU relative abundance between samples. Principal coordinate analysis (PCoA) [18] was used to visually display the information in the distance matrices (i.e. Jaccard, JSD), to explore the relationship between periodontal status and overall fungal composition. Permutational multivariate analysis of variance (PERMANOVA) [19] of the distance matrices was used to examine statistically whether overall fungal composition differed by periodontal status.

### Differential abundance by periodontal status

The 8493 OTUs were agglomerated to 5 phyla, 19 classes, 40 orders, 63 families, 81 genera, and 154 species. We filtered the data to include only taxa present in at least 10% of participants, leaving 4 phyla, 13 classes, 19 orders, 25 families, 30 genera, 49 species, and 916 OTUs for this analysis. We used Wilcoxon rank-sums tests to compare relative abundance of these taxa between the periodontal disease group and oral health group, and the two-sample test of proportions to compare carriage (i.e. presence/absence) of the taxa between the groups.

### Statistical principles

All statistical tests were two-sided, and a *p*-value < 0.05 was considered statistically significant. All analyses were conducted using R (version 3.2.0).

## Results

### Participant characteristics

The demographic characteristics of the participants (*n* = 30) in this study are shown in Table 1. The participants were on average 67.0 ± 7.8 (mean ± SD) years old, and 46.7% were male. Participants with periodontal disease (*n* = 15) did not differ significantly from participants with oral health (*n* = 15) in age, sex distribution, BMI, years since quitting smoking, floss frequency, or tooth brush frequency (all *p* > 0.15).

**Table 1:** Demographic characteristics of participants

	Periodontal disease (*n* = 15)	Oral health (*n* = 15)	P-value^a^
Male, n (%)	7 (46.7)	7 (46.7)	1.00
Age, mean ± SD (range)	68.4 ± 8.9 (49–80)	65.6 ± 6.5 (51–71)	0.34
BMI category,^b^ n (%)			0.33^c^
Under- or normal-weight	7 (46.7)	11 (73.3)	
Overweight	6 (40.0)	4 (26.7)	
Obese	1 (6.7)	0 (0.0)	
Missing	1 (6.7)	0 (0.0)	
Years since quitting smoking, mean ± SD (range)	31.4 ± 8.8 (16–49)	33.1 ± 10.8 (11–46)	0.49
Floss frequency, n (%)			0.15^c^
None	0 (0.0)	4 (26.7)	
1–4 times per week	7 (46.7)	5 (33.3)	
5–7 times per week	5 (33.3)	2 (13.3)	
≥ 8 times per week	3 (20.0)	3 (20.0)	
Missing	0 (0.0)	1 (6.7)	
Tooth brush frequency, n (%)			0.59
1 time per day	1 (6.7)	3 (20.0)	
2 times per day	9 (60.0)	8 (53.3)	
3 times per day	5 (33.3)	3 (20.0)	
≥ 4 times per day	0 (0.0)	1 (6.7)	
Swollen, inflamed, or bleeding gums, n (%)	2 (13.3)	NA^d^	
Amount of permanent teeth lost, n (%)			
1–2 teeth	4 (26.7)	NA^d^	
3–4 teeth	4 (26.7)		
5–9 teeth	2 (13.3)		
≥ 10 teeth	5 (33.3)		

^a^From Wilcoxon rank-sum test for continuous variables and Fisher’s exact test for categorical variables

^b^Under- or normal-weight: BMI < 25 kg/m^2^; Overweight: 25 ≤ BMI < 30 kg/m^2^; Obese: BMI ≥ 30 kg/m^2^

^c^P-value calculated after removing missing values

^d^Not applicable as these were exclusion criteria for oral health group

### Fungal community characteristics

**Fig. 1:**
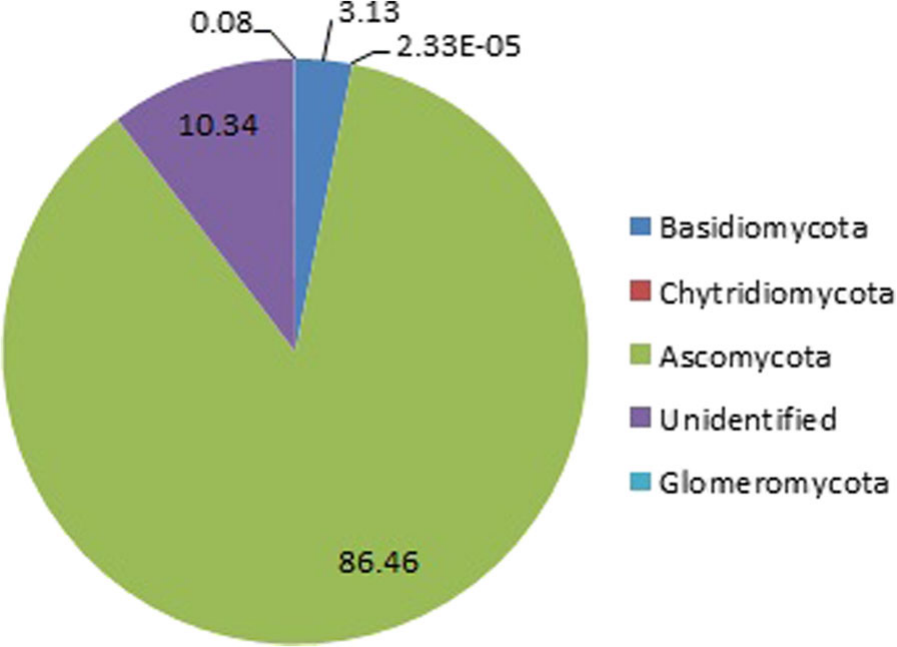
Pie chart showing the frequency (%) of sequence reads belonging to each fungal phylum from 30 oral wash samples

Across all samples, we observed at least 5 phyla (Ascomycota, Basidiomycota, Glomeromycota, Chytridiomycota, and unclassified), 81 genera, 154 species, and 8943 OTUs. The majority of sequence reads in the samples were from the phylum Ascomycota (86.5%), 10.3% were unidentified, 3.1% were from phylum Basidiomycota, 0.1% from Glomeromycota, and 2.3e-05% from Chytridiomycota (Fig. 1). The number of OTUs per participant ranged from 77 to 2262.

**Fig. 2:**
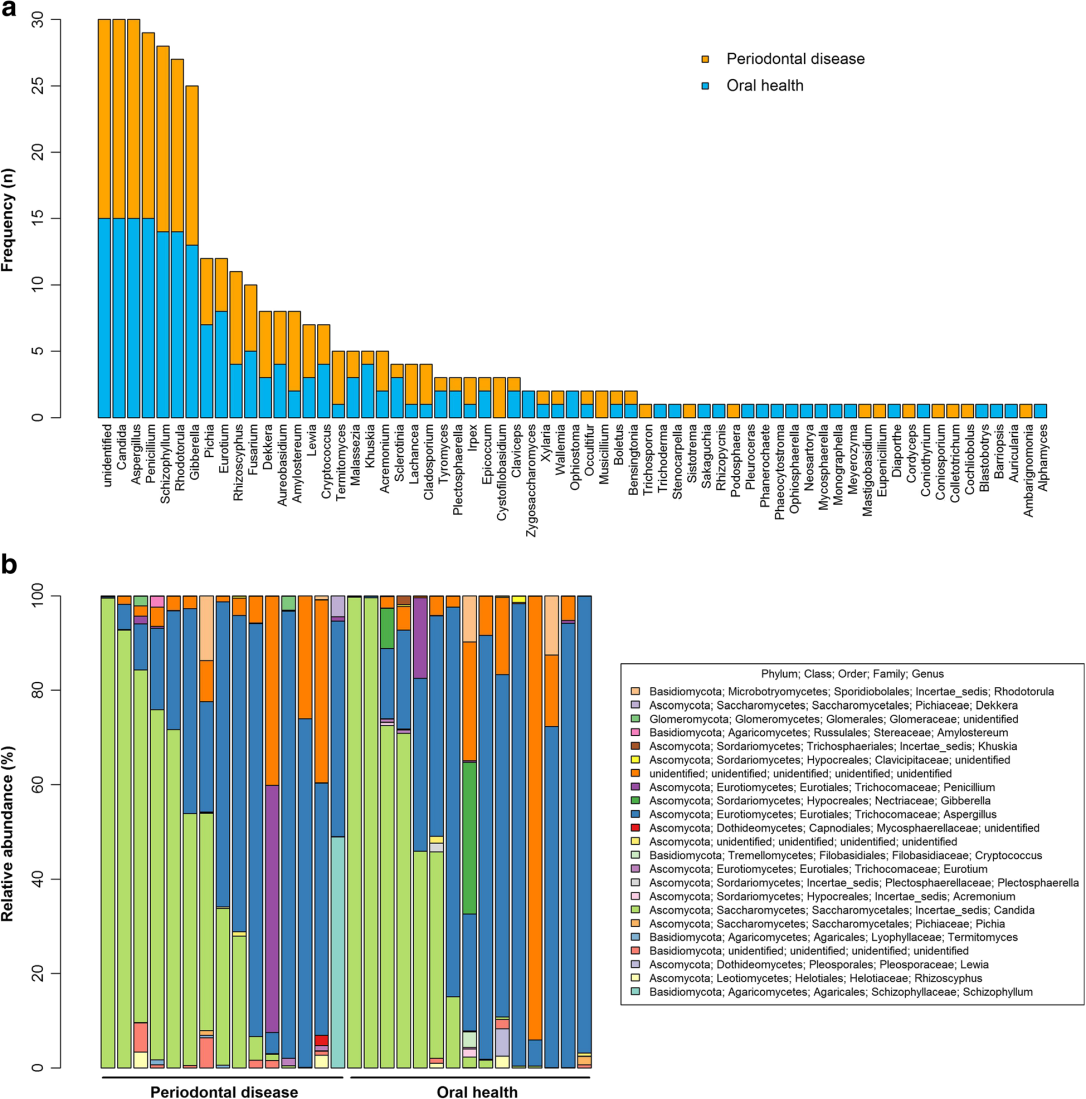
**a** Frequency and **b** relative abundance of fungal genera in oral wash samples. In (**a**), all unidentified genera were grouped into one bar, and all other genera are shown individually. In (**b**), only the 23 most abundant genera are shown

**Fig. 3:**
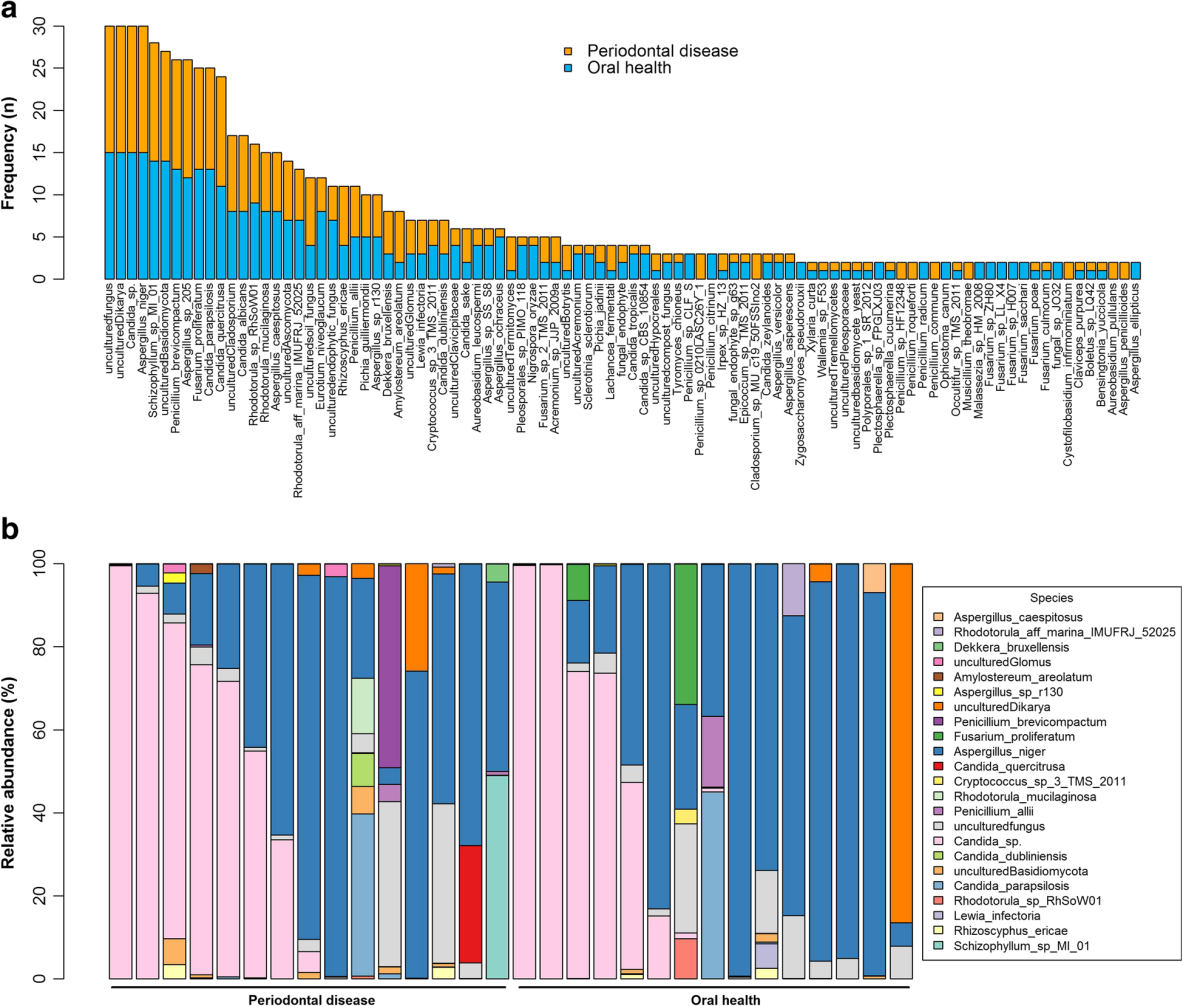
**a** Frequency and **b** relative abundance of fungal species in oral wash samples. In (**a**), all unidentified species were grouped into one bar, and all other species present in at least two people are shown individually. In (**b**), only the 23 most abundant species are shown

Of the 81 genera, 33 were present in only one person, and the remaining 48 were shared by two or more participants. *Candida* and *Aspergillus* were the most frequently observed (isolated from 100% of participants), followed by *Penicillium* (97%), *Schizophyllum* (93%), *Rhodotorula* (90%), and *Gibberella* (83%) (Fig. 2a). *Candida* and *Aspergillus* were also the most highly abundant genera in the samples (median relative abundance = 21% and 44%, respectively), though there was substantial variation in abundance of these two genera across samples (Fig. 2b). At species level, three species were observed in all 30 participants (uncultured *Dikarya*, *Candida* sp., and *Aspergillus niger*), and all participants also had unidentified fungus (Fig. 3a). The most abundant species in the samples were *Aspergillus niger*, unidentified fungus, and *Candida* sp. (median relative abundance = 44%, 2.5%, and 1%, respectively) (Fig. 3b).

### Overall fungal diversity in relation to periodontal disease

**Fig. 4:**
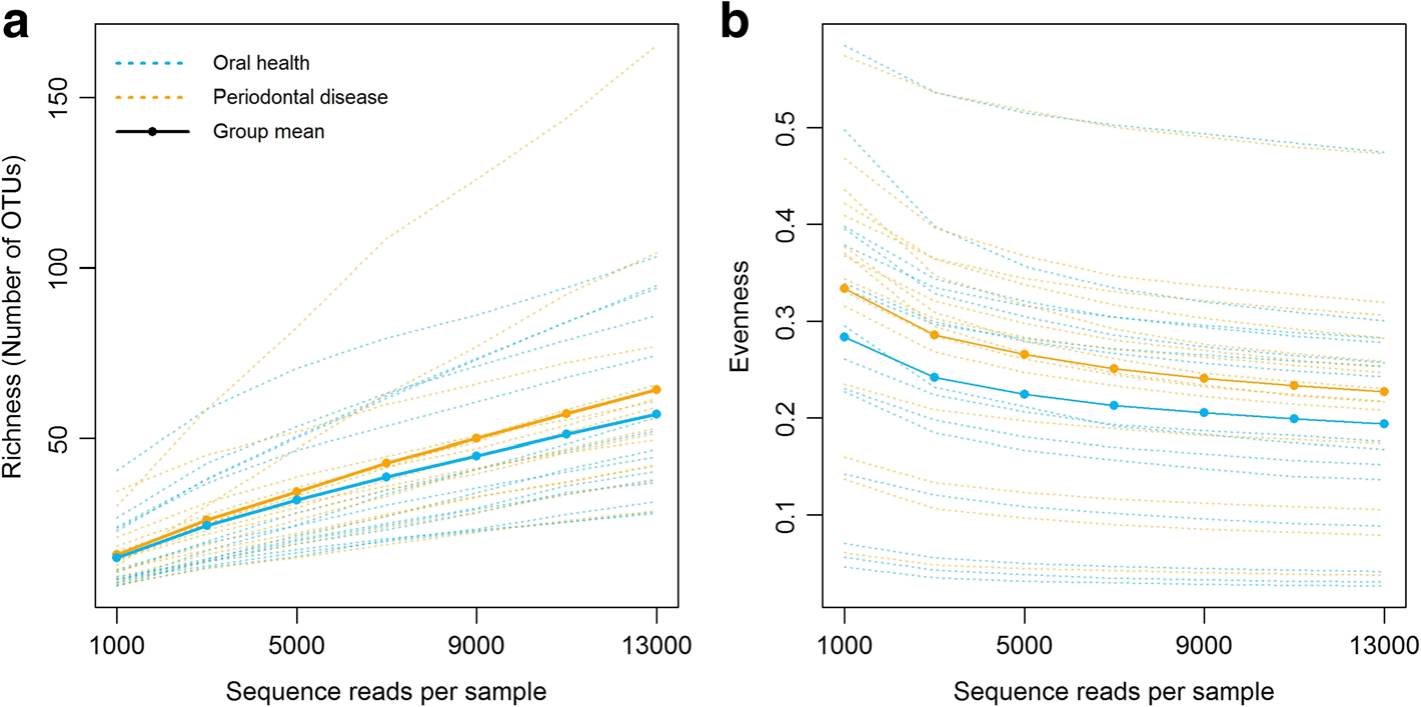
Rarefaction curves of (**a**) richness and (**b**) evenness. These indices were calculated for 100 iterations of rarefied OTU tables, and the average over the iterations was taken for each participant. Means are shown for the periodontal disease and oral health groups

**Fig. 5:**
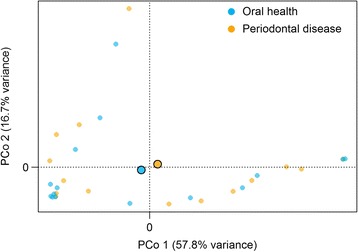
Principal coordinate analysis of the Jensen-Shannon Divergence (JSD). The first two coordinates are plotted. *Shapes outlined* in *black* represent centroids for periodontal disease and oral health groups

The number of fungal OTUs per participant tended to be higher in the periodontal disease group than in the oral health group, as did the evenness of the fungal community (Fig. 4); however, these diversity indices did not differ significantly between the two groups (*p* = 0.40 and *p* = 0.44, respectively). Likewise, the overall composition of the fungal community at OTU level did not differ significantly between the periodontal disease and oral health groups (Jaccard index *p* = 0.83, JSD *p* = 0.91) (Fig. 5).

### Fungal taxa in relation to periodontal disease

**Fig. 6:**
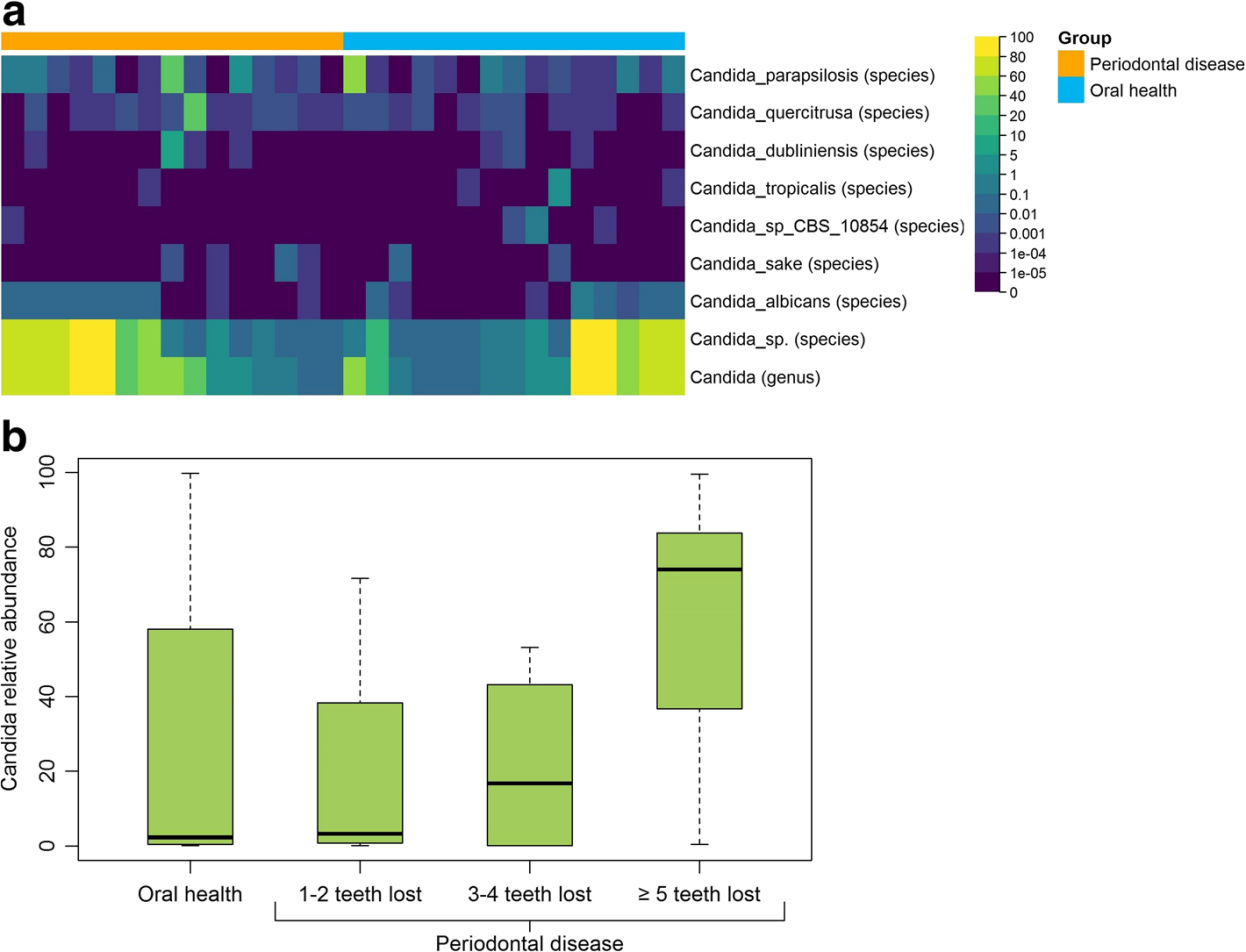
**a** Relative abundance of genus *Candida* and *Candida* species in participants with periodontal disease or oral health and **b** relative abundance of genus *Candida* by number of permanent teeth lost

The genus *Candida*, previously implicated in periodontal disease [11, 12], was more abundant in participants with periodontal disease than participants with oral health (median relative abundance = 33.2% and 2.2%, respectively), though this difference was not significant (*p* = 0.52) (Fig. 6a, Table 2). The same was generally true for *Candida* species (Fig. 6a, Table 2). Interestingly, within the periodontal disease group, the median relative abundance of genus *Candida* also increased with increasing number of permanent teeth lost (1–2 teeth lost: 3.2%; 3–4 teeth lost: 16.6%; ≥5 teeth lost: 73.9%; *p* = 0.11) (Fig. 6b); however, sample size for this analysis was very small. While we did not observe significant differences between participants with periodontal disease and participants with oral health for phylum-species level taxa, we found 33 OTUs that were differentially abundant between the groups at *p* < 0.05 (Table 3); 14 from *Aspergillus niger*, three from *Candida albicans*, and 14 from *Candida* sp. We did not observe any group difference patterns (i.e. same direction of association) for the OTUs from *Aspergillus niger* or *Candida albicans*, though the OTUs from *Candida* sp. all had higher relative abundance in participants with periodontal disease than participants with oral health. These OTUs were no longer associated with periodontal disease after adjustment for multiple comparisons (false discovery rate [20] method).

**Table 2:** *Candida* and periodontal disease

	Carriage, %			Relative abundance, median (IQR)		
	Periodontal disease (*n* = 15)	Oral health (*n* = 15)	*P*-value	Periodontal disease (*n* = 15)	Oral health (*n* = 15)	*P*-value
Candida (genus)	100	100	NA	33.2 (0.9, 72.7)	2.2 (0.4, 58)	0.52
*Candida albicans* (species)	60	53.33	1	7.5e-04 (0, 0.04)	2.87e-04 (0, 0.01)	0.56
Candida dubliniensis (species)	26.67	20	1	0 (0, 1.82e-04)	0 (0, 0)	0.6
*Candida parapsilosis* (species)	80	86.67	1	1.2e-03 (5.8e-04, 0.1)	7.8e-04 (4e-04, 0.07)	0.69
Candida quercitrusa (species)	86.67	73.33	0.65	7.6e-04 (4.6e-04, 1.9e-03)	4.9e-04 (7.8e-05, 1.2e-03)	0.35
*Candida sake* (species)	26.67	13.33	0.65	0 (0, 1.2e-04)	0 (0, 0)	0.48
Candida sp. (species)	100	100	NA	5.0 (0.09, 72.2)	0.8 (0.08, 56.7)	0.49
Candida sp_CBS_10854 (species)	6.67	20	0.59	0 (0, 0)	0 (0, 0)	0.28
*Candida tropicalis* (species)	6.67	20	0.59	0 (0, 0)	0 (0, 0)	0.31

**Table 3:** Fungal OTUs associated with periodontal disease

Phylum; Class; Order; Family; Genus; Species	Carriage, %			Relative abundance, median (IQR)		
	Periodontal disease (*n* = 15)	Oral health (*n* = 15)	*P*-value	Periodontal disease (*n* = 15)	Oral health (*n* = 15)	*P*-value
Ascomycota; Dothideomycetes; Pleosporales; unidentified; unidentified; Pleosporales_sp_PIMO_118 (OTU)	0	26.67	0.11	0 (0, 0)	0 (0, 0.000164)	0.04
Ascomycota; Eurotiomycetes; Eurotiales; Trichocomaceae; Aspergillus; Aspergillus_niger (OTU)	53.33	13.33	0.05	0.000403 (0, 0.00252)	0 (0, 0)	0.03
Ascomycota; Eurotiomycetes; Eurotiales; Trichocomaceae; Aspergillus; Aspergillus_niger (OTU)	33.33	0	0.05	0 (0, 0.000383)	0 (0, 0)	0.02
Ascomycota; Eurotiomycetes; Eurotiales; Trichocomaceae; Aspergillus; Aspergillus_niger (OTU)	40	6.67	0.08	0 (0, 0.00148)	0 (0, 0)	0.03
Ascomycota; Eurotiomycetes; Eurotiales; Trichocomaceae; Aspergillus; Aspergillus_niger (OTU)	26.67	0	0.11	0 (0, 0.000124)	0 (0, 0)	0.04
Ascomycota; Eurotiomycetes; Eurotiales; Trichocomaceae; Aspergillus; Aspergillus_niger (OTU)	26.67	0	0.11	0 (0, 0.000202)	0 (0, 0)	0.04
Ascomycota; Eurotiomycetes; Eurotiales; Trichocomaceae; Aspergillus; Aspergillus_niger (OTU)	26.67	0	0.11	0 (0, 0.000124)	0 (0, 0)	0.04
Ascomycota; Eurotiomycetes; Eurotiales; Trichocomaceae; Aspergillus; Aspergillus_niger (OTU)	0	26.67	0.11	0 (0, 0)	0 (0, 0.000165)	0.04
Ascomycota; Eurotiomycetes; Eurotiales; Trichocomaceae; Aspergillus; Aspergillus_niger (OTU)	0	26.67	0.11	0 (0, 0)	0 (0, 0.000165)	0.04
Ascomycota; Eurotiomycetes; Eurotiales; Trichocomaceae; Aspergillus; Aspergillus_niger (OTU)	0	26.67	0.11	0 (0, 0)	0 (0, 0.000164)	0.04
Ascomycota; Eurotiomycetes; Eurotiales; Trichocomaceae; Aspergillus; Aspergillus_niger (OTU)	0	26.67	0.11	0 (0, 0)	0 (0, 0.000165)	0.04
Ascomycota; Eurotiomycetes; Eurotiales; Trichocomaceae; Aspergillus; Aspergillus_niger (OTU)	0	26.67	0.11	0 (0, 0)	0 (0, 2e-04)	0.04
Ascomycota; Eurotiomycetes; Eurotiales; Trichocomaceae; Aspergillus; Aspergillus_niger (OTU)	0	33.33	0.05	0 (0, 0)	0 (0, 0.000246)	0.02
Ascomycota; Eurotiomycetes; Eurotiales; Trichocomaceae; Aspergillus; Aspergillus_niger (OTU)	0	33.33	0.05	0 (0, 0)	0 (0, 0.000398)	0.02
Ascomycota; Eurotiomycetes; Eurotiales; Trichocomaceae; Aspergillus; Aspergillus_niger (OTU)	20	53.33	0.13	0 (0, 0)	0.000328 (0, 0.000467)	0.02
Ascomycota; Saccharomycetes; Saccharomycetales; Incertae_sedis; Candida; Candida_albicans (OTU)	33.33	0	0.05	0 (0, 0.00019)	0 (0, 0)	0.02
Ascomycota; Saccharomycetes; Saccharomycetales; Incertae_sedis; Candida; Candida_albicans (OTU)	0	26.67	0.11	0 (0, 0)	0 (0, 7.76e-05)	0.04
Ascomycota; Saccharomycetes; Saccharomycetales; Incertae_sedis; Candida; Candida_albicans (OTU)	0	26.67	0.11	0 (0, 0)	0 (0, 7.76e-05)	0.04
Ascomycota; Saccharomycetes; Saccharomycetales; Incertae_sedis; Candida; Candida_sp (OTU)	33.33	0	0.05	0 (0, 0.000215)	0 (0, 0)	0.02
Ascomycota; Saccharomycetes; Saccharomycetales; Incertae_sedis; Candida; Candida_sp (OTU)	26.67	0	0.11	0 (0, 8.41e-05)	0 (0, 0)	0.04
Ascomycota; Saccharomycetes; Saccharomycetales; Incertae_sedis; Candida; Candida_sp (OTU)	26.67	0	0.11	0 (0, 6.56e-05)	0 (0, 0)	0.04
Ascomycota; Saccharomycetes; Saccharomycetales; Incertae_sedis; Candida; Candida_sp (OTU)	26.67	0	0.11	0 (0, 6.56e-05)	0 (0, 0)	0.04
Ascomycota; Saccharomycetes; Saccharomycetales; Incertae_sedis; Candida; Candida_sp (OTU)	26.67	0	0.11	0 (0, 6.56e-05)	0 (0, 0)	0.04
Ascomycota; Saccharomycetes; Saccharomycetales; Incertae_sedis; Candida; Candida_sp (OTU)	26.67	0	0.11	0 (0, 6.56e-05)	0 (0, 0)	0.04
Ascomycota; Saccharomycetes; Saccharomycetales; Incertae_sedis; Candida; Candida_sp (OTU)	26.67	0	0.11	0 (0, 6.56e-05)	0 (0, 0)	0.04
Ascomycota; Saccharomycetes; Saccharomycetales; Incertae_sedis; Candida; Candida_sp (OTU)	26.67	0	0.11	0 (0, 0.000131)	0 (0, 0)	0.04
Ascomycota; Saccharomycetes; Saccharomycetales; Incertae_sedis; Candida; Candida_sp (OTU)	26.67	0	0.11	0 (0, 8.41e-05)	0 (0, 0)	0.04
Ascomycota; Saccharomycetes; Saccharomycetales; Incertae_sedis; Candida; Candida_sp (OTU)	26.67	0	0.11	0 (0, 6.56e-05)	0 (0, 0)	0.04
Ascomycota; Saccharomycetes; Saccharomycetales; Incertae_sedis; Candida; Candida_sp (OTU)	26.67	0	0.11	0 (0, 6.56e-05)	0 (0, 0)	0.04
Ascomycota; Saccharomycetes; Saccharomycetales; Incertae_sedis; Candida; Candida_sp (OTU)	26.67	0	0.11	0 (0, 0.000131)	0 (0, 0)	0.04
Ascomycota; Saccharomycetes; Saccharomycetales; Incertae_sedis; Candida; Candida_sp (OTU)	26.67	0	0.11	0 (0, 6.56e-05)	0 (0, 0)	0.04
Ascomycota; Saccharomycetes; Saccharomycetales; Incertae_sedis; Candida; Candida_sp (OTU)	26.67	0	0.11	0 (0, 0.00019)	0 (0, 0)	0.04
unidentified; unidentified; unidentified; unidentified; unidentified; unculturedfungus (OTU)	33.33	0	0.05	0 (0, 0.000265)	0 (0, 0)	0.02

## Discussion

In the current study characterizing the oral mycobiome of adults with periodontal disease or oral health, we observed a small core set of genera, species, and OTUs that were shared by all or most participants, and a much larger number of genera, species, and OTUs that were shared by few participants or unique to single participants. In particular, the genera *Candida* and *Aspergillus* were shared by all participants and were present at the highest relative abundance in the oral fungal communities on average.

Few studies have characterized the oral mycobiome using comprehensive ITS gene sequencing. Ghannoum et al. reported 85 genera and 101 species present across oral rinse samples from 20 healthy individuals, using ITS sequencing [2]. We have observed a similar number of genera and species in our samples (81 and 154, respectively), in agreement with that study. Additionally, similar genera were identified in ours and the Ghannoum et al. study, albeit at differing frequencies: for example, while we observed *Candida* and *Aspergillus* presence in 100% of participants, they observed *Candida* and *Aspergillus* presence in 75% and 35% of participants, respectively. Findings of our study and that study diverge even further at species level, where the most commonly observed species in our study, *Aspergillus niger*, was not observed at all in the Ghannoum et al. study. A study by Dupuy et al. characterized the oral mycobiome of saliva samples from six individuals using ITS sequencing, and observed high frequency (100%) of genus *Malassezia* [21], as well as high frequency of *Candida*, *Aspergillus*, and others. We have observed the genus *Malassezia* as well, albeit at lower frequency (17%). With so few studies of the oral mycobiome currently published, it is difficult to pinpoint a reason for differences between our study and these other studies; possible reasons include differences in geographical residence of study participants, differences in race/ethnicity, and differences in methodology (i.e. sample types, DNA extraction, sequencing technology, downstream sequence read processing and taxonomic assignment).

We did not observe significant differences in overall oral mycobiome diversity and composition between participants with periodontal disease and participants with oral health, nor did we observe significant differences in phylum through species level taxon relative abundance or carriage between the two groups. We observed 33 OTUs that were significantly differentially abundant between the groups (*p* < 0.05), however because we conducted many comparisons at OTU level (916 tests), these findings may be due to chance. Lack of significant findings may relate to insufficient power, as our sample size was small. It is also possible that oral wash samples do not adequately represent the fungi present in the periodontium, the location where fungi would presumably influence periodontal disease development. A culture-based study of 28 periodontally healthy subjects, 20 subjects with aggressive periodontitis, and 26 subjects with chronic periodontitis, observed that subjects with chronic periodontitis were more likely to be carriers of yeasts, and had a higher degree of yeast colonization, particularly for *Candida albicans*, *Candida dubliniensis*, and *Candida glabrata*, than periodontally healthy subjects [12]. That study examined both mucosal and subgingival sites, and noted that the differences in yeast carriage and abundance were more apparent in the subgingiva than in the mucosa [12]. Another culture-based study also reported higher carriage and abundance of yeasts, including *Candida albicans*, in the subgingiva of 40 chronic periodontitis subjects compared to 20 periodontally healthy subjects [11]. While we observed higher relative abundance of genus *Candida* in oral wash samples from participants with periodontal disease compared to participants with oral health, and a trend of increasing *Candida* in participants with greater number of permanent teeth lost, these differences were not significant. The higher relative abundance of 14 *Candida* sp. OTUs in participants with periodontal disease compared to oral health may indicate that periodontal disease is associated with rare *Candida* types. Nevertheless, the evidence from culture-based studies suggests that subgingival sites may be more appropriate for assessing the relationship of oral fungi with periodontitis.

Ours is the first study to characterize the oral mycobiome in relation to disease using a comprehensive targeted sequencing approach. While small sample size may have limited our power to detect significant associations with periodontal disease, our results suggest that *Candida* species may be more highly abundant in the oral cavity of subjects with periodontal disease, in agreement with previous reports [11, 12]. Whether this may be a cause or consequence of periodontal disease remains to be determined from experimental studies. Interestingly, fungi and bacteria can interact in several ways, including physically, chemically, and metabolically, to influence microbial survival, colonization, and biofilm formation [6, 22]. *Candida albicans* was previously shown to enhance *Porphyromonas gingivalis* invasion of human gingival epithelial and fibroblast cells in vitro [23], suggesting that *Candida albicans* may contribute to periodontal pathogen initiation or exacerbation of periodontal disease. As the bacterial origins of periodontal disease are well understood [10], future studies should examine interactions between fungal species and bacterial periodontal pathogens in the development and progression of periodontal disease [24]. Such studies may uncover novel therapeutic approaches for the prevention and treatment of periodontal disease.

## Conclusions

In conclusion, we characterized the oral mycobiome in 30 subjects, providing further insight into the fungal make-up of the oral cavity and building upon the characterizations of previous studies by other groups [2, 21]. Though strong signatures related to periodontal disease were not observed, this may relate to the small sample size and use of samples collected by oral rinse rather than subgingival samples. An additional study limitation is the self-report of periodontal disease status, which can potentially lead to disease misclassification. Additional studies are needed in larger, diverse populations to further establish the core oral mycobiome in health, and relate it to disease states.

## Abbreviations

ITS: Internal transcribed spacer
OTU: Operational taxonomic unit
